# The Spanish Family Quality of Life Scales under and over 18 Years Old: Psychometric Properties and Families’ Perceptions

**DOI:** 10.3390/ijerph17217808

**Published:** 2020-10-25

**Authors:** Anna Balcells-Balcells, Joana M. Mas, Natasha Baqués, Cecilia Simón, Simón García-Ventura

**Affiliations:** 1 School of Psychology, Education and Sport Sciences Blanquerna, Ramon Llull University, 08022 Barcelona, Spain; JoanaMariaMM@blanquerna.url.edu (J.M.M.); natashaBA@blanquerna.url.edu (N.B.); simongv@blanquerna.url.edu (S.G.-V.); 2 School of Psychology, Autonomous University of Madrid, 28049 Madrid, Spain; cecilia.simon@uam.es; 3 School of Psychology, Abat Oliba CEU University, 08022 Barcelona, Spain

**Keywords:** Family Quality of Life, intellectual and developmental disabilities, Family Quality of Life Scale, measure, Spanish Family Quality of Life Scales, CdVF-ER > 18, CdVF-ER < 18

## Abstract

Background: Family quality of life (FQoL), just like individual quality of life, has become a priority outcome in the policies and services received by persons with intellectual and developmental disabilities (IDD) and their families. Conceptualizing, measuring, and theorizing FQoL has been the object of investigation in recent decades. The goal of this paper is to present a revision of the Spanish Family Quality of Life Scales, the CdVF-E < 18 and the CdVF-E >18, and describe the FQoL of Spanish families with a member with IDD. Methods: The sample included a total of 548 families with a member under 18 years old and 657 families with a member over 18. Based on an Exploratory Factor Analysis (EFA) firstly and a Confirmatory Factor Analysis (CFA) secondly, the two scales’ psychometric properties were explored. Results: The CdVF-ER < 18 and the CdVF-ER > 18 comprise 5 dimensions, containing 35 and 32 items, respectively, and they show good validity and reliability. The families obtained a high FQoL score, although some differences exist between the dimensions on which families with children under and over 18 score highest and lowest. Conclusion: The characteristics of the revised scales facilitate their use by professionals, administrations, and services.

## 1. Introduction

In the late 1970s and early 1980s, the concept of quality of life (QoL) emerged as a reference framework in the field studying individuals with intellectual and developmental disabilities (IDD) [1,2]. 

Increasing recognition that the QoL of the individual with IDD is closely related to their environment and, particularly, the role of the family in their life as a natural context of development [1,3,4,5,6] has established Family Quality of Life (FQoL) as an essential outcome for the services assisting these individuals [1,7,8,9,10,11,12]. In this sense, one of the most up-to-date FQoL definitions, and one of the most widely cited, is the one by Zuna et al. [13] (p.262), which highlights the relationship between the individual and the family in FQoL perception: “a dynamic sense of well-being of the family, collectively and subjectively defined and informed by its members, in which individual and family-level need to interact.”

Indeed, in the last 20 years, FQoL has triggered researchers’ interest in the field of IDD. Over these years, the studies conducted by researchers regarding FQoL have focused on: (1) conceptualizing FQoL; (2) developing measurement instruments and adapting them to different cultural contexts; and (3) finding out about families’ QoL to prepare a theory explaining the construct while identifying how FQoL behaves against specific predictors [14,15].

The early papers focused on conceptualizing FQoL, and they intended to discover what families understood as FQoL and identify its main components [16,17,18,19]. The studies in the second group are devoted to preparing measurement instruments to assess the construct. In many cases, these studies went hand in hand with the conceptualization studies where FQoL dimensions were identified [3,19,20].

The most internationally renowned instruments to measure FQoL according to Samuel et al. [21] are the Beach Center Family Quality of Life Scale (Beach Center FQoL) [22], originally developed in the USA, and the International Family Quality of Life Scale (FQOL-2006) [20,23], created by Australian, Canadian, Israeli, South Korean, and Taiwanese researchers. Both instruments were designed for families with members with IDD of all ages. They have been the object of investigation in many studies in order to optimize their tools [24,25,26], validate them in social and cultural contexts, e.g., Italy, Taiwan, China, Greece, Canada, Singapore, Spain [27,28,29,30,31,32,33,34,35,36], and studying their psychometric properties in more depth [20,37,38,39].

In parallel, other instruments have been developed to measure FQoL. Some of these are the Families in Early Intervention Quality of Life (FEIQoL) [40], initially prepared by McWilliam and Casey [41] and focusing on the stage of early intervention (children from 0–6 years old), the Spanish Family Quality of Life Scale for families with a child with IDD under 18 years old (CdVF-E < 18), and the Spanish Family Quality of Life Scale for families with a member with IDD over 18 years old (CdVF-E > 18) [42]. The last two are the object of study of this article.

Although all the scales above measure families’ FQoL perception, here is how they differ: (a) sphere of application or the population they are intended for (whether culture, population, or ages of members with IDD); (b) characteristics of the instrument (closed-ended questions or a combination of open- and closed-ended questions); (c) the dimensions they assess (see Appendix A). As regards the latter aspect, it should be noted that each scale is made of different FQoL dimensions that allow researchers to measure the construct globally. Some of these dimensions are similar in different scales, although others are quite different [39,40]. 

Based on the different instruments, various studies focused on describing FQoL in specific populations from Spain, Colombia, Australia, or Brazil [40,43,44,45,46]. Other intended to describe what FQoL families perceived according to their individual and/or collective characteristics [24,47,48,49,50] or as a result of receiving certain supports or intervention practices [14,51,52,53,54,55,56].

These studies brought about the construct’s theorization, i.e., defining an explicative theory that helped to holistically understand how FQoL factors and predictors are related. In this sense, Zuna et al. [13] made an explicative proposal in which they identified four factors that influence the families’ FQoL: (a) the family unit; (b) the characteristics of the individuals who form the family; (c) performance; and (d) the system. The authors proposed middle-range theories that account for how these variables relate to one another to determine FQoL and urged researchers to validate these proposals through empirical research. They also requested an improvement in the existing instruments to measure FQoL, claiming that they are still not sensitive enough to perceive changes attributable to the factors comprised in their theoretical proposal Along the same lines, the Families Special Interest Research Group of the International Association for the Scientific Study of Intellectual and Developmental Disabilities [57] has repeatedly claimed that improving the FQoL instruments appears as one of the challenges of international research on FQoL. Either new proposals must be constructed, or the existing instruments must be refined to move forward, regarding the use of such instruments both in research and practice [13,15,22]. Consequently, just as happened with the individual QoL model, FQoL, understood as an outcome, has become a quality indicator of the policies, services, and programs received by individuals and families with IDD [22]. Likewise, it is also the framework to guide the planning of support and services for a better fit with families’ actual needs. For administrations and services, using FQoL measurement instruments has in fact become an essential element to learn about the global situation of these families’ lives.

The Spanish Family Quality of Life Scale for families with a child with IDD under 18 years old (CdVF-E < 18) and the Spanish Family Quality of Life Scale for families with a member with IDD over 18 years old (CdVF-E > 18), the two Spanish Family Quality of Life Scales (CdVF-E), were developed by the research group “Disability and Quality of Life: Educational Aspects” in 2013 [42]. These scales were the result, on the one hand, of the qualitative inquiry conducted to conceptualize FQoL in Spain and, on the other, a quantitative process that included trialing an early pilot version of the two scales as well as their final version in Spain.

There are two CdVF-E scales: one for children up to 18 years of age (CdVF-E < 18) and another for adults over 18 (CdVF-E > 18). The needs of individuals with IDD change substantially over time and, consequently, so do the needs of their families and the impact on their QoL perception. In Spain, the age of majority is 18. Reaching that age involves several changes; among them, the degree of disability and the family’s financial compensation are revised. In addition, new choices open up: education, independent life, and employability. These choices vary according to each person’s support needs and possibilities. In order to be more sensitive to such life changes and their possible impact on FQoL, the authors considered it advisable to have two different instruments [42]. 

The first psychometric approach conducted in Spain regarding the CdVF-E < 18 and the CdVF-E > 18 scales provided a one-dimensional measure. The items measured FQoL as a whole although they were constructed from seven conceptual areas: Emotional well-being, Family interaction, Health, Financial well-being, Organization and parental skills, Family accommodation, and Social inclusion and participation [44]. 

Since their creation, both scales have been subjected to adaptation and validation in other countries. In Costa Rica, Cordero [58] confirmed the multi-dimensionality of the CdVF-E < 18 in a 0–6-year-old population. More specifically, the author identified eight dimensions (Financial Well-being, Family Accommodation, Parental Abilities, Family Climate, Autonomy of the Member with Disability, Health, Formal Supports, and Informal Supports). However, Bitencourt [59] adapted and validated the CdVF-E > 18 scale in Brazil, and still found it to be one-dimensional.

Even though—as pointed out—numerous papers have in recent years have focused on the construction, validation, or study of tools to measure FQoL [14,20,22,23,24,25,26,27,28,29,30,31,32,33,34,35,36,37,38,39], authors agree on the need to continue to devote efforts to optimizing their internal structures and revising how they work in different populations in different contexts (socio-demographic, cultural, etc.) [25,29,31,39,40]. In this sense, after applying the CdVF-E < 18 and the CdVF-E > 18 scales for 5 years, we deemed it appropriate to revise them both and identify their internal structures for research [44,60] and intervention purposes in the Spanish context [61]. 

The purpose of that revision was twofold: on the one hand, to improve their efficiency by shortening them and thus improving the answer choices; and on the other, to study their psychometric properties according to reliability and validity criteria. More FQoL-sensitive instruments will provide better knowledge of the families’ realities and, therefore, their needs will be better met.

Consequently, the goals of this article are: (a) to present the psychometric properties and final features of the new version of the scales named Spanish Family Quality of Life Scale for families with a child with IDD under 18 years old. Revised version in 2019 (CdVF-ER < 18) and Spanish Family Quality of Life Scale for families with a member with IDD over 18 years old. Revised version in 2019 (CdVF-ER > 18); and (b) to describe the FQoL perception of the families who participated in the study.

The ultimate objective is indeed to provide two tools to assess the FQoL of Spanish families that are more efficient and offer better psychometric guarantees, and which adapt to the period of life the families are undergoing. Therefore, the current paper should be understood as an extension of the measurement studies conducted in the field of FQoL thus far.

## 2. Materials and Methods

### 2.1. Type of Study

An instrumental type of study is presented for the first goal, classified among empirical studies as a quantitative methodology. The guidelines of the Standards for Educational and Psychological Testing [62] were observed in its preparation, since they compile different trustworthy procedures and strategies to guarantee a test’s reliability and validity. The International Test Commission Guidelines [63] were also observed, which feature the general principles for the use of tests.

For the second goal, a descriptive study was conducted in order to present the participating families’ FQoL perceptions.

### 2.2. Participants

A total of *N* = 548 families with one underage member with IDD participated in the study as well as *N* = 657 families with an adult member. They came from 13 Spanish Autonomous Communities. Table 1 shows the main characteristics of the individual who answered the scale and the member with IDD.

The inclusion criteria for the participants were: (a) having a child with Intellectual or Developmental Disability diagnosed by the official social centers in each Autonomous Community and, (b) being a Spanish resident.

### 2.3. Instrument

The scales submitted for revision originated from the Spanish Family Quality of Life Scale, (CdVF-E) [42], both the version for families with an underage member with IDD (CdVF-E < 18), comprising 61 items, and the version for adults (CdVF-E > 18), comprising 67 items. Both were one-dimensional scales and featured seven areas interpreted as tendencies: Emotional well-being, Family interaction, Health, Financial well-being, Organization and parental skills, Family accommodation, and Social inclusion and participation. The answers were of the frequency type according to a 5-point Likert scale where 1 meant *never* and 5 *always*, plus one *it does not apply (NA)* choice. The scales’ reliability was acceptable, with an internal consistency in agreement with a 0.96 Cronbach’s alpha.

### 2.4. Procedure

Firstly, Plena Inclusión (Confederación Española de Organizaciones en favor de las Personas con Discapacidad Intelectual, one of the most important associations in Spain for persons with a disability and their families), through their different organizations, administered the CdVF-E scales [42] to the families. This process took place over 5 years in 13 Autonomous Communities of Spain. Regarding ethical safeguards, the participants were provided with information on the project as well as confidentiality safeguards. Secondly, the results were kept anonymously in a centralized computer database managed by Plena Inclusión. Prior to data storage, the families had to give their consent for their information to be added to the organization’s computer file so they could be treated, analyzed, and published without prejudice to their rights, according to the applicable current law within the Ley Orgánica 15/1999, Protección de Datos de Carácter Personal de España (Spanish Personal Data Protection Regulation). The ethical principles of the Spanish Psychological Association were also observed.

In total, information was gathered from 878 families of underage individuals with IDD and 2730 families with an adult member with IDD, all of them coming from the different organizations federated with Plena Inclusión. However, scales with over 20% of unanswered items were discarded from this study.

### 2.5. Dataset

Descriptive analyses of the items. The authors studied the descriptive statistics of each item to discover how each behaved. Additionally, they explored those that had answered *it does not apply* (NA) in 20% of the sample and considered them as lost data. As a result of those analyses, 11 items from the under-18 scale and 7 items from the adults scale were removed. Moreover, the answer *it does not apply* (NA) was discarded, given the families’ tendency to choose this answer frequently.

Factor analyses. In this stage of the study, the authors explored the internal structure of both scales, and their reliability.

For this purpose, the samples were randomly divided into two datasets (under and over 18 years old). With one half of the sample, sample A, the authors conducted reliability and validity analyses by means of an Exploratory Factor Analysis (EFA), and assessed the results both empirically and theoretically. The extraction method used was the principal component analysis and the oblimim oblique rotation method. Later, in order to refine and empirically confirm the structure resulting from the EFA, a confirmatory factor analysis (CFA) was conducted with the other half of the sample, sample B, in both sets. The parameter estimation method used was Maximum Likelihood (ML) in the case of the under-18 scale, and Robust Maximum Likelihood (MLR) for the over-18 scale. To assess the global fit model, the authors calculated the Chi-square index (χ^2^), associated likelihood *p* and the degrees of freedom (*df*), and the Root Mean Square Error of Approximation (RMSEA), in addition to the confidence intervals and the Standardized Root Mean Square Residual (SRMR). Regarding the incremental fit measures, they assessed the Normed Fit Index (NFI), the Non-Normed Fit Index (NNFI), or the Tucker Lewis Index (TLI), and the Comparative Fit Index (CFI). To increase each factor’s consistency and explore alternative structures, Modification Indices (MI) were explored.

Study of reliability and normative scores. Lastly, the authors provided evidence of reliability through Cronbach’s alpha coefficient, and calculated the central tendency and dispersion based on the mean, the standard deviation, the minimum, the maximum, and the percentiles, for the total and for each of the dimensions of both scales (CdVF-ER < 18 and CdVF-ER > 18).

Data analysis was conducted using the Statistical Package for the Social Sciences (SPSS) software, version 21.0 and MPlus software, version 7.4 [64].

## 3. Results

As a result of the scale refinement process through EFA, 15 items were removed from the under-18 scale (CdVF-ER < 18) and 28 items from the adults scale (CdVF-ER > 18). The EFA result for the CdVF-ER < 18 suggested a 5-factor solution that explained 50.83% of the variance and comprised 35 items. For the CdVF-ER > 18, a 5-factor solution was identified that explained 49.69% of the variance and comprised 34 items.

Below are the reliability and validity indices of the CdVF-ER < 18 and the CdVF-ER > 18 scales as well as their final characteristics after conducting the CFA, and the descriptive statistics of the population under study, in accordance with the two main goals of this study.

### 3.1. Reliability and Validity Indices and Final Characteristics of the Scales

Regarding the first objective of this study, presenting the psychometric properties identified in the new version of the scales, the validity indices for the CdVF-ER < 18 scale yielded highly acceptable results. As regards the absolute fit measurements, Chi-square Index (χ^2^), and associated likelihood (959.989: *p* < 0.001), they indicated that the model proposed did not fit the data. However, to minimize the impact of the sample size, the magnitude was considered (χ^2^/gl): at 1.79, this presented an acceptable value [65]. Regarding the RMSEA Index, the value was 0.052, which is indicative of a close fit [66], and the associated confidence intervals were (0.047–0.058). SRMR, with a value of 0.052, indicated an acceptable fit [67]. Concerning the incremental fit measurements, TLI was 0.934 and CFI 0.926, indicative of a good fit [68]. Table 2 shows the Goodness of fit indices for the final model of the CdVF-ER < 18 and the CdVF-ER > 18 scales.

Regarding the latent variables, the Family resources dimension related significantly to the Family climate (0.445, *p* = 0.000), Financial well-being (0.4, *p* = 0.002), and Emotional stability (0.499, *p* = 0.000) dimensions. As for item reliability, standardized factor loadings yielded values between 0.120 (item 5) and 0.691 (item 3).

The reliability of the CdVF-ER < 18 scale, measured through Cronbach’s alpha, was 0.91, and it ranged between 0.73 (Emotional stability and Family adaptation) and 0.86 (Family climate and Financial well-being) in each of the family dimensions. Table 3 presents these results.

The final solution for the CdVF-ER < 18 scale comprises 5 FQoL dimensions and 35 items divided as follows: Family climate (12 items), Financial well-being (8 items), Emotional stability (8 items), Family adaptation (11 items), and Family resources (9 items).

As regards the CdVF-ER > 18 scale, the absolute fit measurement was 6.28 (χ^2^/*gl*), and the RMSEA index value was 0.040. The associated confidence intervals were 0.034 and 0.046 and the SRMR value was 0.051. Concerning the incremental fit measurements, TLI was 0.898 and CFI 0.911. Table 2 shows the Goodness-of-fit indices of the final model of both scales, the CdVF-ER < 18 and the CdVF-ER > 18.

All the latent variables yielded a significant relationship to one another (*p* = 0.000), where the most significant relationships were found between Family support for the person with IDD and Family climate (0.933), and between Family organization and Family climate (0.730).

On the other hand, the standardized loadings ranged between 0.115 (item 18) and 0.730 (item 22), while the limit of acceptable values is 0.30 according to some authors [69].

Regarding the reliability of the CdVF-ER > 18, Cronbach’s alpha value for the total was 0.89 and the values for each area ranged between 0.81 for the Financial well-being area and 0.70 for Family support for the person with IDD. Table 4 presents Cronbach’s alpha for the total and for each of the CdVF-ER > 18 scale’s dimensions.

The final solution for the CdVF-ER > 18 scale comprised 5 FQoL dimensions and 32 items divided as follows: Family climate (14 items), Autonomy of the person with IDD (12 items), Financial well-being (5 items), Family organization and functioning (11 items), and Family support to the person with IDD (8 items).

Given the existence of correlation between certain dimensions of both scales, some items load in more than one dimension at the same time [70]. An example of the items for both scales can be found in Appendix B.

### 3.2. Descriptive Statistics

With regard to the second objective, describing the FQoL perception of the families who participated in the study, Family quality of life perceived by the families with an underage child yielded a global mean of 3.99 (*SD* = 0.49). Regarding the FQoL dimensions, the highest score was Family adaptation (*M* = 4.15), followed by Family resources (*M* = 4.15). The lowest scores were obtained in the Financial well-being (*M* = 3.73) and Emotional stability dimensions (*M* = 3.81). Table 5 presents the scores obtained.

As regards the family quality of life perceived by the families with an adult member, the global mean obtained was 4.03 (*SD* = 0.48). Concerning the FQoL dimensions, the highest score was Family organization and functioning (*M* = 4.15), followed by Financial well-being (*M* = 4.09). The lowest scores were obtained for Autonomy of the person with IDD (*M* = 3.75) and Family support to the person with IDD (*M* = 3.74). Table 6 presents the scores obtained.

## 4. Discussion

The present study pursued two goals: firstly, to show the psychometric properties and final features of the CdVF-ER < 18 and the CdVF-ER > 18 scales; and, secondly, to approach the way families perceive their FQoL. 

As regards the first goal, similarly to the studies that have validated other FQoL scales, both for underage [22,25,27,32,34,36,39] and adult populations [31], the psychometric properties of the scales presented satisfactory reliability (with Cronbach’s Alpha over 0.90) and validity indices. This guarantees that they can be used in the Spanish context for research and intervention purposes. With regard to the final features of the scales, like most existing scales that measure FQoL based on a series of dimensions, the present scales have shown they both consist of five dimensions, respectively, thus reflecting the multifactorial nature of FQoL [1,20,71]. On the one hand, the dimensions identified for the CdVF-ER < 18 were: Family climate, Financial well-being, Emotional stability, Family adaptation, and Family resources. On the other, the dimensions identified for the CdVF-ER > 18 were: Family climate, Autonomy of the person with IDD, Financial well-being, Family organization and functioning, and Family support to the person with IDD. Accordingly, two dimensions appeared in both scales: Family climate and Financial well-being.

The dimensions identified in both scales are not conceptually related in an accurate manner to the dimensions of the other existing FQoL scales, as is also the case among the scales developed thus far, and as proved by some authors [38,40]. Although some dimensions have similar names and assess very similar aspects, in other cases similar indicators are included in a different dimension or have been grouped into new dimensions. For this reason, trying to relate the dimensions of the CdVF-ER < 18 and the CdVF-ER > 18 scales to those of other scales is only carried out as an example of the coherence of some dimensions and indicators between scales, and can help explain each dimension’s conceptual meaning.

To this effect, below are the descriptions of the dimensions found in both scales and their conceptual relationship with the dimensions of the rest of the FQoL scales presented in the introduction. Appendix A lists and defines the Beach Center FQoL dimensions [22] and the Family relationships of FQOL-2006 [20,23] and FEIQoL [40]. The first two scales concern families with a member with IDD of any age, whereas FEIQoL [40] focuses on families with children from 0–6 years old.

In this sense, the authors will first focus on the dimensions that the CdVF-ER < 18 and the CdVF-ER > 18 scales have in common, and then on the remaining ones.

Family climate refers to the quality of the relationship between the several members of the family (supporting and accepting each other as they are; having good communication; trusting each other; staying united and facing difficulties, etc.). This dimension agrees with Family interaction in the Beach Center FQoL [22], and Family relationships of FQOL-2006 [20,23] and FEIQoL [40], as can be seen in Appendix A. The Financial well-being dimension—related to the family’s financial and material situation in facing the whole family’s needs and the specific needs of the member with IDD—could be related to the Physical and Material Wellbeing dimensions of the Beach Center FQoL [22], or the Financial Wellbeing of FQOL-2006 [20,23].

As regards the other dimensions of the CdVF-ER < 18, we can observe that Emotional stability bears certain resemblance to Emotional wellbeing of the Beach Center FQoL [22], given that both are related to feelings of peace, quiet, personal projects, life as a couple, etc.

The rest of the CdVF-ER < 18 dimensions, such as Family resources and Family Adaptation, only share a few items with some of the dimensions of other FQoL scales and thus they cannot be likened, given that an important number of items of the scales presented in this paper have been grouped differently. However, we did find that Family resources–which focuses on both the family’s emotional and service resources (formal supports) in facing their relative’s needs—shares some resemblance with the Disability-related support dimension of the Beach Center FQoL [22], Support from other people and Support from disability-related services of the FQOL-2006 [23], and Access to Information and Services of FEIQoL [40]. For its part, Family Adaptation—understood as those aspects related to the family’s acceptance and adaptation to their relative’s disability, their needs and characteristics—resembles the Parenting dimension of the Beach Center FQoL [22]. 

The same applies to the rest of the CdVF-ER > 18 scale’s dimensions. For example, Family organization and functioning—which concerns whether home chores and responsibilities are shared, as well as the care, attention, and education of the person with IDD—presents some items that also appear in the Parenting dimension of the Beach Center FQoL [22], among others. The dimension Family support to the person with IDD concerns the resources the family has to help their relative with IDD in solving daily life matters, managing their love and sex life, and planning their future. This dimension shares a few indicators with the following dimensions: Disability-related support of the Beach Center FQoL [22] and Support from other people and Support from disability-related services of the FQOL-2006 [20,23]. Lastly, yet another dimension identified in the CdVF-ER > 18 scale is Autonomy of the person with IDD. This dimension focuses on the perception families have regarding the autonomy of their relative with IDD according to their age; including their participation in community activities. Indicators of this dimension can be found in different dimensions of other scales, for instance, Community and civic involvement of the FQOL-2006 [20,23]. 

The second goal of the study was to describe the FQoL perception of the families who participated in the study. In agreement with other studies, the results showed a high global perception of FQoL for both types of families–under and over 18 years old—[14,44,60,72,73]. Similarly to other studies [60,72,74], families with an adult member show a higher FQoL perception than families with an underage member.

As regards FQoL perception, families with an underage member with IDD perceive they have better FQoL in relation to the Family adaptation and Family resources dimensions. On the other hand, the dimensions with the lowest scores are Financial well-being and Emotional stability.

Bearing in mind that Family adaptation encompasses items related to family accommodation, social inclusion and participation, and parental skills and organization, the results of the current study show that Spanish families perceive that they have adapted well to the needs of their relative’s disability, and that they have integrated them well into their family dynamics. This agrees with other studies [73]. According to the authors, it makes sense that the families who scored high on this dimension also value the family’s resources positively. This dimension includes not only the family’s formal support, but also the capability they perceive they have in facing the challenges of their relative’s development. As regards more formal supports, the results presented here match those of other studies conducted in Spain [14,40] as well as in other countries [75].

As concerns the dimensions scored lowest by the families in this study, one of the dimensions that proves vulnerable for Spanish families is Financial well-being, in agreement with other studies [14,42,48,60,76]. More specifically, the families in this study indicate their lack of financial resources to face the future with confidence or to occasionally indulge themselves. However, the results obtained for Emotional stability—in light of other studies—are ambiguous. On the one hand, the results of this study agree with those of other studies conducted with other scales, like Balcells-Balcells et al. [14], Davis and Gavidia-Payne [51], or Summers et al. [54], where the Emotional well-being dimension was one of the least satisfactory for the families. On the other hand, the current results are not consistent with those obtained by some other studies conducted in Spain and with the scale’s original version [42,44,60]. In the authors’ opinion, the cause of this discrepancy lies in the fact that, in the current study, unlike in those previously mentioned, the FQoL measure used (CdVF-ER < 18) features statistically identified dimensions.

Concerning the families with an adult member with IDD, the dimensions with the highest scores are Family organization and functioning and Financial well-being, whereas the lowest scores go to Autonomy of the person with IDD and Family support to the person with IDD.

Similarly to the families with an underage child, these families perceive they have good Family organization and functioning, which implies that not only do they divide their house chores but also the functions and responsibilities related to their relative with IDD. This includes some aspects of emotional well-being. Unlike the families with an underage child with IDD, the families with adult children scored as high on the Financial well-being dimension. This would explain why the Family functioning dimension was high, among other reasons. The difference between the assessment of families with an underage and an adult member was also found in Giné et al. [60].

On the other hand, the dimensions with the lowest scores were Autonomy of the person with IDD and Family support to the person with IDD. In the authors’ opinion, these results are explained by the fact that over 80% of the family members with IDD show a degree of disability between moderate and high, which involves important support needs. Likewise, the fact that the dimension Family support to the person with IDD obtains the lowest scores may be related to the degree of support the individuals need. It should be noted that this dimension enquires about aspects concerning the love and sex life of the person with IDD, and their plans for their future. These are complex subjects for the families and they frequently delegate them to professionals in the services their relative attends. In this sense, both Bertelli [77] and Jokinen and Brown [72] found that support-related dimensions were the least satisfactory for the families.

To sum up, the scales under revision in this article, the CdVF-ER < 18 and the CdVF-ER > 18, present good psychometric properties to measure the FQoL perception of families with an underage and an adult member. Moreover, the scales revised have certain strengths, which the authors would like to highlight. On the one hand, they allow researchers to measure the construct’s multi-dimensionality based on 5 dimensions by scale; and on the other, they do so with shorter scales (with fewer items), and without the *it does not apply* choice, which facilitates the answering the scale for the families and applying and interpreting it for the professionals. Improved efficiency encourages administrations and services to use it more easily. The new format of the scales (fewer items and changes in the type of answer) make it easier to fill it out, thus reducing barriers when completing them: the time needed, and understanding the type of answer. Therefore, the revision of the scales met the two challenges that triggered this revision: the scale’s multi-dimensionality and having more efficient scales that continue to provide relevant information to plan the necessary resources to improve the families’ QoL. That had been, since the onset of the scales, an essential premise. In that way, they maintain the value of focusing on one specific evolution stage of the families (with underage or adult children) and consequently, they facilitate the planning of the most adequate support for each moment/situation in life.

### Limitations

Most of the participants in this study were mothers. Wang et al. [78] proved there was no difference between fathers and mothers regarding FQoL perception. In consequence, most researchers sum up mothers’ and fathers’ perceptions globally, regardless of their role in the family [22,25,27,31,39]. Although some studies claim that fathers’ perceptions do not differ significantly from those of mothers, Vanderkerken et al. [79] argue that it is still important to investigate the impact of the participation of one or more family members on the FQoL. There are no studies in Spain regarding the differences in the FQoL perceptions of fathers and mothers. It would be advisable to replicate this type of study to confirm this statement in the Spanish context. 

This study presents another limitation, which is that the data was collected by the Plena Inclusión organization. Having the support of organizations to collect data is an advantage as concerns the number of participants the study has. But it also has some disadvantages, namely: (a) an important number of scales had over 20% unanswered items and, therefore, could not be used for the analyses; and (b) certain demographic information was not collected, for example, socio-economic level or place of residence. This information would have helped the authors interpret some aspects in more depth.

One relevant aspect that the current study also shares with most FQoL studies is the high scores the families give to the indicators that allow researchers to value the construct. Several colleagues like Chiu et al. [25] attribute this fact to the instruments being distributed by the children’s assigned professionals. In any case, this is an aspect to bear in mind when analyzing the scores, both from the perspective of research and intervention. They should be complemented, especially in the latter case, with qualitative techniques such as interviews.

## 5. Conclusions and Implications for Research and Practices

The results obtained in this study as regards the psychometric properties and final features of the scales lead to the following conclusions:

- Since the first FQoL scales were created, the authors of the current paper have confirmed the reach of measuring FQoL, both for research (to improve knowledge) and intervention purposes (adjusting supports to the families’ QoL).
- The results presented in this paper provide evidence that the CdVF-ER >18 and the CdVF-ER < 18 are both reliable and valid for measuring FQoL in Spain throughout the lifetime of the member with IDD. The CdVF-ER > 18 and the CdVF-ER < 18 scales have proved useful and easier to use than previous scales (in time and implementation). Furthermore, they are formed by several FQoL factors (5 dimensions each), which facilitates result interpretation and good validity and reliability.

As for the FQoL perception of the families who participated in this study, it can be argued that:

- Spanish families with a member with IDD have a high perception of their FQoL although families with an adult member show a higher FQoL perception than families with an underage member.
- Families with an underage member with IDD give a higher score to the Family adaptation and Family resources dimensions, whereas Financial well-being and Emotional stability obtained a lower score. For the families with an adult with IDD, Family organization and functioning and Financial well-being were the best-rated dimensions, better than Autonomy of the person with IDD and Family support to the person with IDD, which had with the lowest scores.

As far as research is concerned, it would be interesting to administer the scales to find out how they work on heterogeneous groups (ages, regions, family characteristics) and adapt them and validate them in other social and cultural contexts. This will allow researchers to explore whether the factors identified behave similarly in different places with different characteristics. 

Using scales for research purposes would also allow investigators to shed light on certain FQoL-related questions and on the support the services provide, more specifically to assess the impact of the services and the efficacy of the programs on FQoL more efficiently, and to evaluate the role of quality in the relationships between the professionals and the families in FQoL, etc.

At the same time, the CdVF-ER > 18 and the CdVF-ER < 18 scales may have positive practical implications at different levels. Firstly, on a micro level, the information obtained from these scales—along with other data—will allow the professionals who work closely with the families to adapt their intervention better to each family’s circumstances and needs. Secondly, at a program-service level, having data on how the families in the organization perceive their current FQoL will allow coordinators and directors to offer and/or design supports better adapted to their population. Finally, on a policy level, they should encourage policies that focus on fostering FQoL. Likewise, given that FQoL has also proved to be an important service outcome, the authors consider that the information provided by these scales should not only help design more adequate proposals, but also measure the impact of those proposals.

In short, being able to measure, and therefore, move forward in conceptualizing and discovering families’ situations to provide more adequate support is not only going to help improve the general family context, but also each family member’s situation. Schalock et al. [4], in their theoretical QoL proposal, defend the position that family-related aspects, such as FQoL, moderate the QoL of individuals with IDD. More specifically, the authors argue that the chances for the development of individuals with IDD are motivated by the family’s characteristics. This justifies the services’ current challenge: improving the QoL of individuals with IDD and also that of their families.

## Figures and Tables

**Table 1:** Demographic characteristics of the family members who answer the scale and the person with intellectual and developmental disabilities (IDD).

		Families with One Member with IDD			
		Under 18 (*N* = 548)		Over 18 (*N* = 657)	
Characteristics		%	n	%	n
Sex of the person who answers					
	Male	23	126	33.5	220
	Female	75.9	416	65.1	428
Relation to the person with IDD					
	Mother	83	455	47.9	315
	Father	13.9	76	25.3	166
	Sibling	0.2	1	23.3	153
	Other	1.8	10	2.7	18
Age of the person who answers (year)					
	Mean	41.52		56.91	
	Age range	21–70		23–88	
Sex of the person with IDD					
	Male	66.6	365	57.5	378
	Female	31.9	175	4.3	265
Age range (years)					
	0–6	33.6	184	-	-
	7–12	39.8	218	-	-
	13–18	24.8	136	-	-
	19–40	-	-	69.6	457
	41–60	-	-	25.4	167
	>61	-	-	1.4	9
Degree of disability ^1^					
	Not recognized (15–33%)	5.7	31	0.5	3
	Mild (33–64%)	42.7	234	10.5	69
	Moderate (65–74%)	21	115	44.3	291
	Severe (>75%)	20.1	110	42.9	282
	In process	2	11	0.2	1
	Not requested	6.2	34	-	-
IDD-associated impairments					
	Autism Spectrum Disorder (ASD)	42	211	6	28
	Hearing impairment	2	10	7.5	35
	Language disorder	15.9	80	13.5	63
	Attention deficit disorder (ADHD)	9.6	48	4.1	19
	Health	12	60	16.3	76
	Conduct disorders	5.8	29	11.4	53
	Visual impairment	6	30	13.5	63
	Physical disability	17.1	86	21.7	101
	Mental disorder	2	10	21.9	102
	Other	26.1	131	-	-
Main service attended by the person with IDD					
	School	55.8	306	4	26
	Vocational training	3.3	18	28.9	190
	Job	0.2	1	18.1	119
	Daycare center	0.4	2	33.2	218
	Leisure	0.4	2	0.8	5
	Residence	0.5	3	9.4	62
	Early intervention	24.5	134	-	-
	Other	12.2	67	-	-

^1^ The degrees of disability were defined according to the scales (%) provided by the Spanish Government.

**Table 2:** Goodness of fit indices for the final model of the CdVF-ER < 18 and CdVF-ER > 18 scales.

Goodness Indices	CdVF-ER < 18 Model	CdVF-ER > 18 Model
χ^2^	959.989(537)	3117.430(496)
*p*	<0.001	<0.001
RMSEA	0.052	0.040
RMSEA (90%)	(0.047–0.058)	(0.034–0.046)
TLI	0.934	0.898
CFI	0.926	0.911
SRMR	0.052	0.051

*X*^2^, Chi-square Index; RMSEA, Root Mean Square Error of Approximation; TLI, Tucker Lewis Index; CFI, Comparative Fit Index; SRMR, Standardized Root Mean Square Residual.

**Table 3:** Cronbach’s alpha of each dimension of the CdVF-ER < 18 scale.

Dimensions	Number of Items ^1^	Cronbach’s Alpha
Total	35	0.91
Family climate	12	0.86
Emotional stability	8	0.73
Financial well-being	8	0.86
Family adaptation	11	0.73
Family resources	9	0.79

^1^ It should be noted that a number of items are repeated in different dimensions.

**Table 4:** Cronbach’s alpha of the total and of each of the CdVF-ER > 18 scale’s dimensions.

Dimensions	Number of Items ^1^	Cronbach’s Alpha
Total	32	0.89
Family climate	14	0.75
Autonomy of the person with intellectual and development disabilities (IDD)	12	0.79
Financial well-being	5	0.81
Family organization and functioning	11	0.77
Family support to the person with IDD	8	0.70

^1^ It should be noted that a number of items are repeated in different dimensions.

**Table 5:** Descriptive statistics of the total and each family quality of life dimension for the CdVF-ER < 18.

	M	Min	Max	SD
Family quality of life (FQoL) total	3.99	2.26	5.00	0.49
Family adaptation	4.25	2.18	5.00	0.48
Family resources	4.15	1.89	5.00	0.54
Family climate	4.08	2.17	5.00	0.58
Emotional stability	3.81	1.63	5.00	0.61
Financial well-being	3.73	1.13	5.00	0.73

M = Mean; Min = Minimum; Max = Maximum; SD = Standard Deviation.

**Table 6:** Descriptive statistics of the total and each family quality of life dimension for the CdVF-ER > 18.

	M	Min	Max	SD
FQoL Total	4.03	1.00	5.00	0.48
Family organization and functioning	4.15	1.00	5.00	0.54
Financial well-being	4.09	1.00	5.00	0.78
Family climate	4.08	1.00	5.00	0.48
Autonomy of the person with IDD	3.75	1.00	5.00	0.64
Family support to the person with IDD	3.74	1.00	5.00	0.72

M = Mean; Min = Minimum; Max = Maximum; SD = Standard Deviation.

## Appendix A

**Table A1:** Dimensions of the Beach Center FQoL [22], FQOL-2006 [20,23], and FEIQoL [39] and their definitions.

Beach Center FQoL [22]	- Family interaction: relationships among family members. - Parenting: activities that adult family members do to help children grow and develop. - Emotional well-being: the aspects of family life that address the emotional needs of family members. - Physical/material well-being: the aspects of family life that address the physical needs of family members. - Support for the family member with a disability: informal and formal support to benefit the family member with a disability.
FQOL-2006 [20,23]	- Health of family: sometimes one or more members of a family have health problems and these problems affect the other members of the family. - Financial well-being: coping financially. Individual members of the family earn different amounts of money and have different financial needs. - Family relationships: general atmosphere or feeling that is usually present in the family. - Support from others: families sometimes get practical and emotional support from a variety of other people, such as relatives, friends, neighbors and others. - Support from disability services: support received from disability-related services to the member with IDD or the family as a whole. - Influence of values: the influence of personal, spiritual, religious, and cultural values on the family. - Careers and preparing for careers: this regards the adult’s work life or the child’s life in learning. - Leisure and Community involvement: the family’s leisure and recreation activities.
FEIQoL [39]	- Family Relationships: this regards the families’ perceptions of communication within the family, parenting, relationships with extended family, and participation in social activities. - Access to Information and Services: this focuses on the families’ knowledge of child development, managing challenging behaviors, specific demands of their child, and their access to resources in their community. - Child Functioning: this regards the families’ perceptions of the child’s engagement, independence, and social relationships in daily routines. - Overall Life Situation: this regards the families’ perceptions of the fulfillment of family needs in areas such as family health status, family economy, and employment.

## Appendix B

**Table A2:** Dimensions, number and examples of the CdVF-ER < 18 and the CdVF-ER > 18 scales’ items.

Scale	Dimension	Number of Items	Item Example
CdVF-ER < 18	Family climate	12	All my family members show love and affection towards each other.
	Emotional stability	8	All our family members carry out our life projects (personal and professional).
	Financial well-being	8	My family can cover the cost of basic needs (food, clothing, etc.).
	Family adaptation	11	My family adapts to the needs of the relative with IDD.
	Family resources	9	My family feels well treated by the health professionals.
CdVF-ER >18	Family climate	14	All my family members show love and affection towards each other.
	Autonomy of the person with IDD	12	The relative with IDD can manage physically autonomously.
	Financial well-being	5	My family can cover the cost of basic needs (food, clothing, etc.).
	Family organization and functioning	11	In my family we share the chores and responsibilities related to the relative with IDD in a balanced way.
	Family support to the person with IDD	8	My family searches for existing resources and support to improve their quality of life.
